# Approaching to Calibration-Free Ion Detection Based on Thin Layer Coulometry with Ultrathin Ion-Selective Membranes

**DOI:** 10.1021/acsmeasuresciau.4c00069

**Published:** 2024-12-10

**Authors:** Yujie Liu, Gastón A. Crespo, María Cuartero

**Affiliations:** † Department of Chemistry, School of Engineering Science in Chemistry, Biochemistry and Health, KTH Royal Institute of Technology, SE-100 44 Stockholm, Sweden; ‡ UCAM-SENS, Universidad Católica San Antonio de Murcia, UCAM HiTech, Avda. Andres Hernandez Ros 1, 30107 Murcia, Spain

**Keywords:** Cathodic Voltammetry, Thin-Layer Coulometry, Ultrathin Ion-Selective Membrane, Charge Transfer, Ion Transfer, Poly(3-octylthiophene), Calibration-Free Analysis

## Abstract

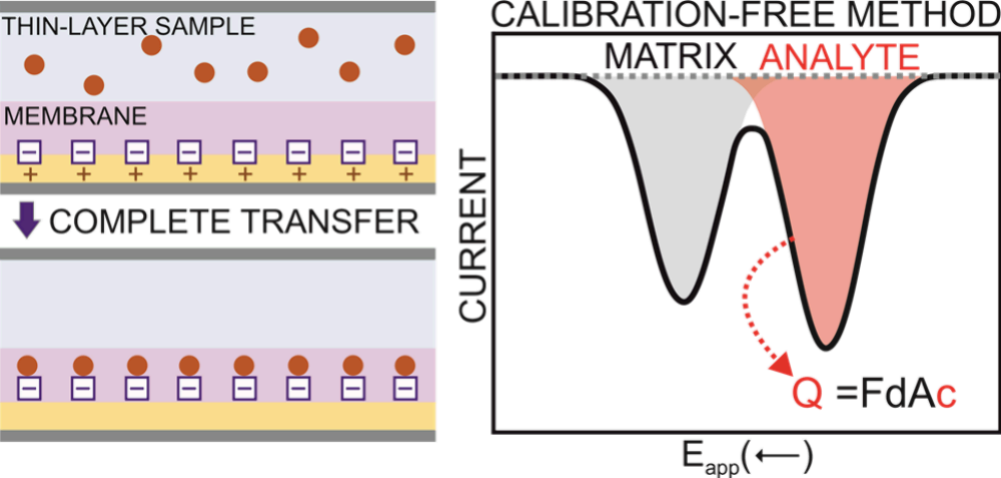

In pursuit of calibration-free all-solid-state ion-selective electrodes (ISEs), we propose a coulometry strategy based on thin-layer samples confined adjacent to the ion-selective membrane (ISM) surface, with the system being controlled under a cathodic potential sweep. The ion-to-electron transducer in the ISE is the conducting polymer poly(3-octylthiophene) (POT), the oxidation state of which changes upon the application of a cathodic sweep and triggers the accumulation of the preferred cation in the ISM. This accumulation is provided of absolute nature (i.e., the cation concentration is totally depleted in the sample) when the capacity of the membrane encompasses the charge of the cation of interest in the sample (K^+^ in this case). As such, the ion exchanger content of the ISM is fixed to 18 μC, being able to accumulate a K^+^ concentration from the solution in the range of 5–40 μM. The charge transfer in the POT film ultimately leads to the K^+^ transfer at the ISM–sample interface, depleting its content in the thin-layer sample with demonstrated efficiency (∼100% at 5 and 1 mV s^–1^). The charge is directly proportional to the corresponding concentration via the Faraday law, constituting the core principle of the calibration-free approach. In essence, there is no need of calibrating the sensor, because the K^+^ concentration can be obtained from the charge by knowing the sample volume with certain precision (volume of 5 μL, with the sample thickness being 100 ± 5 μm). The conceptual innovation introduced in this Letter is accompanied by the validated calibration-free detection of K^+^ in five real samples, demonstrating the plausibility of the approach to contribute to the measurement science field, especially in the direction of fulfilling the gap between benchtop trials and the end users of electrochemical sensors. It is key to put efforts into calibration-free sensors to address real world applications such as point-of-care, wearable sensors for well-being, and environmental in situ monitoring, among others.

In measurement science, a calibration-free approach can be conceived in two ways: as no need for calibration at all or with a minimum calibration step after the fabrication of the analytical device.^1^ With the latter in principle seeming easier to achieve, there are yet a few examples of using this protocol within the field of electrochemical sensors. When a high reproducibility is associated with the manufacturing, a series of sensors from each batch can be typically used to obtain an in-house calibration model that is further applied to the whole batch. Adopting this protocol, the user experience is rather comfortable, as there is no need for additional steps and expertise to obtain the final readings.^2^ Overall, the aim is directly providing the analyte concentration in the sample, and assuring that such information is accurate to make reliable decisions in the field of application.

One strategy toward a calibration-free procedure is to integrate charge-based readouts (i.e., coulometry), allowing for a later concentration calculation via the Faraday law.^3^ Far from being an immediate alternative to put into practice, there are several factors that are essential to achieving the concept of calibration-free, constituting a challenge in today’s electroanalysis directions.^1^ First, the sample volume must be precisely defined; otherwise, the calculations needed for charge-concentration conversion would introduce a significant error in the result. Also, the absoluteness of the process(es) in which the analyte is involved must be ensured, which means that it is ∼100% transported across the sensor phases and/or converted due to the electrochemical input. Moreover, it is convenient to keep the analysis time as short as possible (preferably some seconds but no longer than a few minutes), and hence, sample volume tends to be small to guarantee the total transport/conversion in a reasonable time compatible with analytical purposes. In any case, electrochemistry is the only technique that may allow for a calibration-free principle, in contrast to the rest of analytical techniques portfolio.^1^

A direction to convey the above-mentioned requirements into reality is the combination of coulometry and thin-layer samples (<100 μm in thickness), pursuing the total depletion of the analyte in the sample. Pioneering fundamental investigations were focused on ion-transfer voltammetry at the interface between two immiscible phases.^4−6^ Osakai et al. demonstrated the complete electrolysis for the interfacial transfer of the tetramethylammonium ion, whereas Sanchez-Pedreo and co-workers realized the detection of tetraethylammonium ions. Kihara’s team reported on the coulometric detection of potassium (K^+^), calcium, and magnesium ions. In the three cases, one of the two immiscible phases was a membrane, and the sample was confined in a microfluidic channel (flat cell or tubings).

Advantageously, the coulometry concept can be realized through ion-selective electrodes (ISEs), with the core element being the ion-selective membrane (ISM) containing selective ion receptors, the so-called ionophores. In such a direction, doped polypropylene tubings were suggested for the exhaustive detection of potassium and calcium ions, but also protamine nitrate and nitrite.^7−11^ The application of a constant potential of adequate magnitude generates ion transfer at the interface with the sample, and the ion charge (and concentration) in the sample is obtained from the integration of the corresponding current–time profile. Also, a linear sweep potential can be used, which was recently demonstrated for the calibration-free coulometric detection of K^+^.^12^ To the best of our knowledge, such a work constitutes the first attempt in coulometry to resolve different real samples using all-solid-state ISEs coupled to thin-layer samples. However, the approach was presented with some aspects still to be improved toward a genuine calibration-free technique. Truly, the combination of “calibration-free,” “coulometry,” and “all-solid-state ISEs” is at the time of writing underexplored.

In this Letter, we report on calibration-free all-solid-state coulometric ISEs based on ultrathin ISMs, with the proof-of-concept being the detection of K^+^ in several real samples. Specifically, the charge redout comes from the K^+^ transfer from the sample to the membrane, in contrast to previous reports, which is modulated by the oxidation state of the conducting polymer underlying the membrane, in this case poly(3-octylthiophene) (POT). The absolute accumulation of K^+^ into the membrane is possible owing to the appropriate combination of the exchange capacity of the membrane for positive charges, its thickness, and the sample volume and thickness. Thus, the exchange capacity was fixed to ca. 18 μC by means of the cation exchanger, and this resulted in the detection of micromolar levels (5–40 μM) of K^+^ present in ca. 5 μL of sample volume confined to a thickness of 100 ± 5 μm. This fixed charge is also responsible (together with the applied scan rate) for the peak current levels found in the experiments. On the other hand, the ISM thickness in the nanometer domain is key to visualizing the ion transfer in the form of Gaussian peaks that can be properly analyzed.^12−14^ The system was interrogated under a cathodic regime, obtaining peaks related to the K^+^ transfer that can be treated chargewise. For this, the use of low scan rates (5 and 10 mV s^–1^) was crucial to obtaining well-resolved peaks for the background ions and the K^+^.

Details on reagents, materials, and experiments are provided in the Supporting Information. The POT-ISM was prepared on an ITO electrode cut to the size of 10 mm × 35 mm. A PTFE tape shaped with a circle defined a surface with a diameter of 8 mm in which to electropolymerize a thin layer of POT (thickness ∼50 nm, measured with ellipsometry).^13^ The tape was removed before depositing 25 μL of the cocktail for the ultrathin ISM (thickness of ∼230 nm, measured with ellipsometry) using the spin coater at 1500 rpm for 60 s.^14^ The electropolymerization of POT was carried out by cyclic voltammetry (CV) in a solution containing 0.1 M of 3-octylthiophene and LiClO_4_ in ACN. After degassing with nitrogen for 15 min, two scans from 0 to 1.5 V at a scan rate of 100 mV s^–1^ were performed. The generated POT film was then discharged at 0 V for 120 s to ensure that most of the POT chains are in their neutral state (or basal state, POT^0^). The cocktail to prepare the ISMs contained 20 mg of polyurethane (PU), 20 mg of bis(2-ethylhexyl)sebacate (DOS), 0.8 mg of sodium tetrakis[3,5-bis(trifluoromethyl)phenyl]borate (Na^+^TFPB^–^), and 2 mg of potassium ionophore I (valinomycin) dissolved in 2 mL of tetrahydrofuran (THF).

The ITO-POT-membrane electrode was positioned in a microfluidic cell that allows sandwiching of the thin-layer sample between this and a commercial Pt-SPE (counter-reference electrode for the voltammetric measurements). Details on the design and features of this microfluidic cell were published elsewhere.^12^ The sample was introduced with a peristaltic pump while applying a 1 V potential: the POT film was polarized to avoid the undesired accumulation of cationic species in the membrane (step 1, Figure 1). In essence, POT is in the form of POT^+^ doped with the counteranion of the cation exchanger (TFPB^–^). Then, the pump stops, and the cathodic linear sweep begins from 1.0 to 0 V (step 2, Figure 1), resulting in the POT^+^ in the film being reduced back to POT^0^ (basal state). The TFPB^–^ is liberated in the membrane, and cations present in the solution enter to locally compensate the electroneutrality. Overall, the working mechanism is based on coupled charge and ion transfer processes, as previously demonstrated for analogous systems.^15^

**Figure 1:**
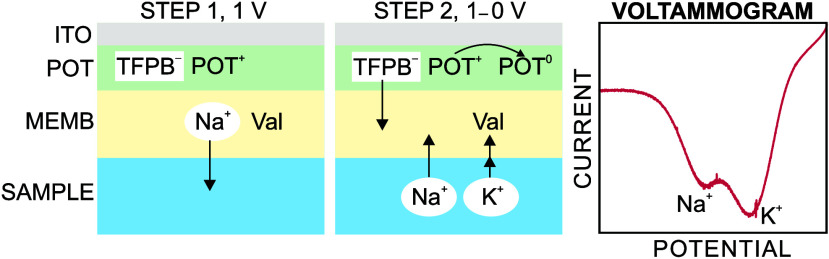
Scheme of the working mechanism adopted for the coulometry analytical measurements of K^+^. MEMB = membrane. Val = Valinomycin.

Notably, the cation that is preferably extracted from the solution to the membrane is K^+^, because of the presence of the K^+^ ionophore. However, other cations in the sample (e.g., Na^+^) are also transferred, with ratios depending on the selectivity profile of the membrane together with the concentration of each cation in the sample solution. Ideally, the extraction of each cation manifests in a separate cathodic peak at different potential windows, with the K^+^ one appearing first (i.e., more positive potentials) because of its more energetically favorable conditions (see Figure 1), and totally separated from the peak associated with the matrix cations.^13,14^ It is here anticipated that the degree of separation of such peaks depends indeed on the scan rate, with the need for providing enough time for each extraction to occur.

Figure 2 shows the results obtained for a solution containing increasing K^+^ concentrations (from 0 to 40 μM) in 10 mM NaCl background electrolyte at scan rates of 100 and 25 mV s^–1^. Notably, in view of the analytical application with real samples, the NaCl background was selected to encompass the presence of other cations in the matrix. It is here anticipated that the transfers of such cations, when possible and at an appropriate scan rate, manifest all in the same peak, whose charge depends on the analyte concentration and position on the cations’ concentration in the sample. This effect has been already described for analogous systems, e.g., for silver detection.^16^

**Figure 2:**
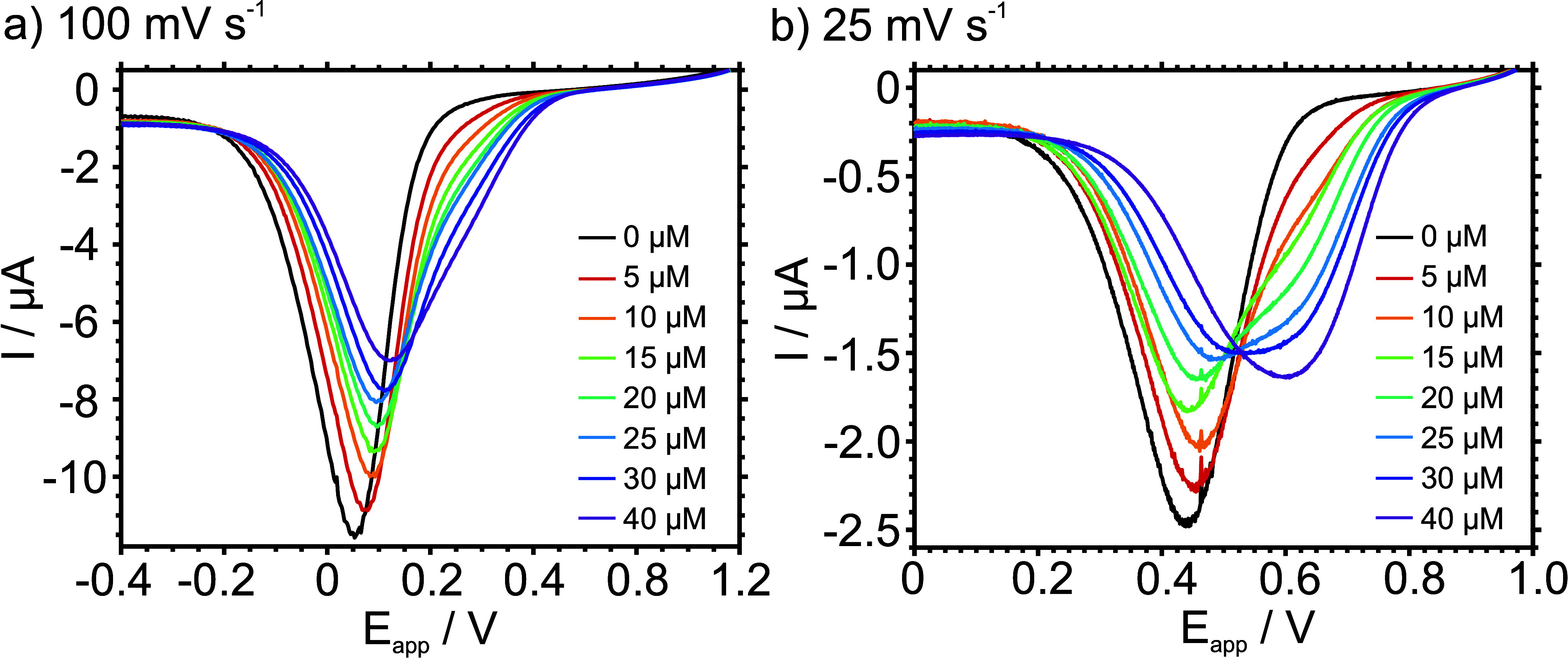
Cathodic peaks observed at increasing concentrations of KCl in 10 mM NaCl background solution at a scan rate of (a) 100 mV s^–1^ and (b) 25 mV s^–1^.

At the described conditions, only one peak was observed for all the K^+^ concentrations measured at 100 mV s^–1^ (Figure 2a), becoming broadened and shifting to more positive potentials (from 50 mV to 150 mV) once K^+^ is incremented. With the naked eye, it seems like a shoulder appears at ca. 0.3 V from the 20 μM, with this being not so conclusive. In contrast, a different profile was observed for the slower scan rate (25 mV s^–1^Figure 2b). The increasing K^+^ concentration resulted in the appearance of a new peak at 650 mV. Thus, this first peak was assigned to K^+^, and that in the potential window of 450–500 mV was assigned to Na^+^ present in the background. It was not until the 15 μM concentration when the K^+^ extraction from the sample to the membrane started to become evident, first as a shoulder, then as a broadening of the original Na^+^ peak (at 25 μM), and finally as a separate peak (40 μM). This outcome suggested that the decrease in the scan rate promotes the accumulation of K^+^ in the membrane.

Further decreasing the scan rate to 5 mV s^–1^ produced even better definition and separation of the Na^+^ and K^+^ peaks. Figures 3a–c displays triplicate results (i.e., using three identical ITO-POT-membrane electrodes) when increasing the KCl concentration in 10 mM NaCl background. The K^+^ peak evidenced from the 5 μM, increasing in terms of current and charge magnitude with increasing K^+^ concentration, in parallel to the decreasing of Na^+^ until it disappeared. While at the beginning the Na^+^ is more prominent than that for K^+^, they become almost equal at a K^+^ concentration of 15 μM, and from this point, the K^+^ peak overcomes, being the only one at the 40 μM concentration.

**Figure 3:**
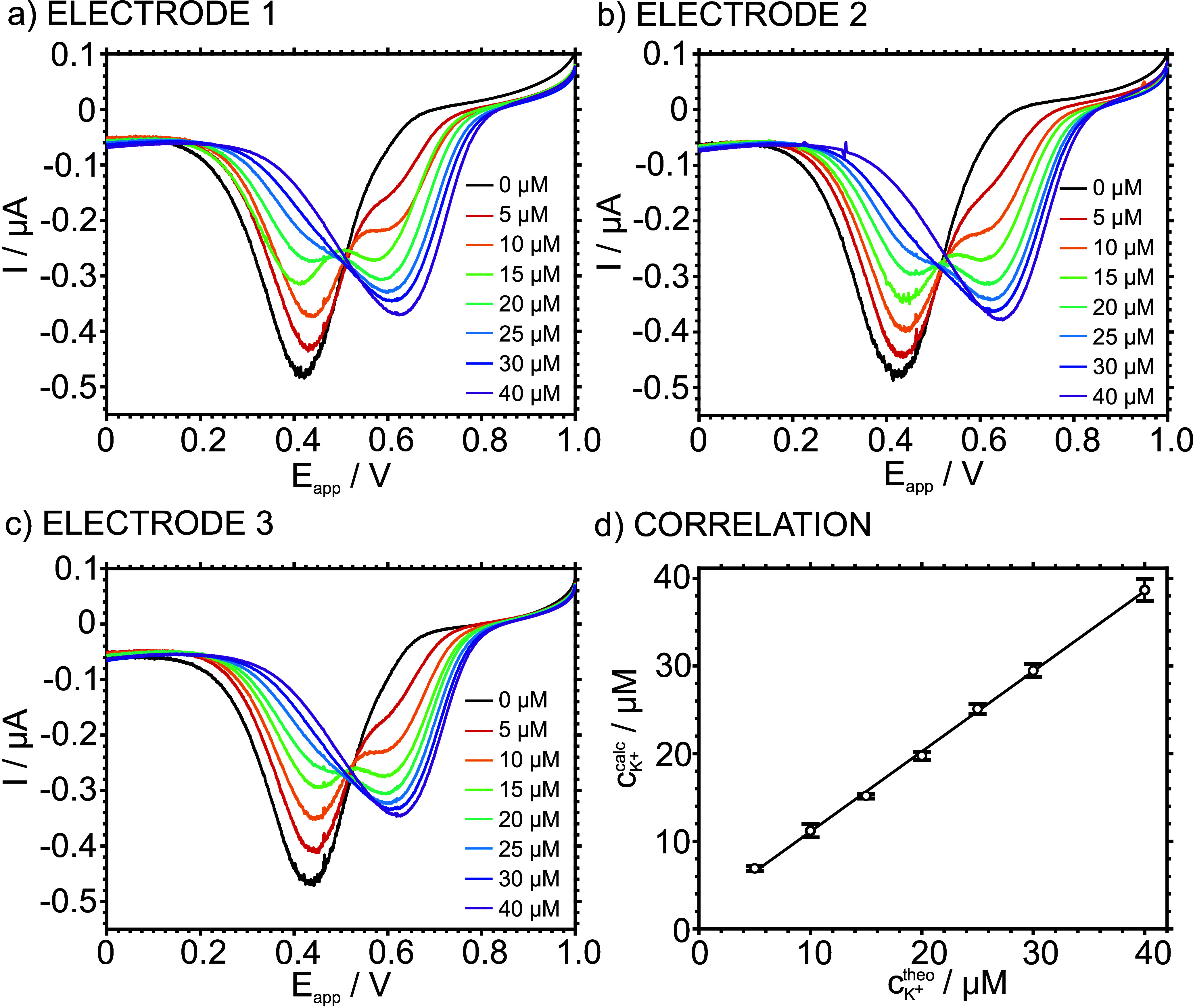
(a–c) Cathodic peaks observed at increasing concentrations of KCl in 10 mM NaCl background solution at a scan rate of 5 mV s^–1^ using three identical ITO-POT-membrane electrodes. (d) Correlation between the calculated and theoretical K^+^ concentration in the sample (*n* = 3 electrodes).

The charge under each voltammetric peak was calculated with baseline correction followed by peak deconvolution via Gaussian fitting, using Matlab R2023a software.^12^ Then, the corresponding K^+^ concentration was obtained applying the Faraday law, considering a sample volume of 5 μL.^12^Figure 3d shows the correlation between the K^+^ concentration calculated by this method and the theoretical values considering the prepared solutions. The data presented a Pearson coefficient of 0.9995, higher than a threshold of 0.95 (to discern the existence or absence of correlation) and thus ensuring a positive correlation. Remarkably, the three electrodes presented very similar results (variation coefficient of ca. 3% for both the peak current and charge, respectively, in the entire K^+^ concentration range), enhancing the possibility of applying the measurement technique as pure calibration-free, even without an initial post-manufacturing calibration.^2^

Regarding the range at which the correlation is found (i.e., from 5 to 40 μM), it is indeed within our expectations. The exchange capacity of the membrane was calculated to be 18 μC, which translates to a K^+^ concentration of about 37.5 μM. Accordingly, the higher concentration that can be supported by the membrane being close to 40 μM, the upper limit of the linear range. From such a level, it is expected that the membrane changes its working behavior from diffusion-control-based (the peak increases with the ion analyte concentration) to thin-layer-based (the peak shifts the potential according to the Nernst principle), as previously demonstrated.^17^ Pursuing a coulometry procedure, what interests us is the first mode, since more charge will accumulate in the membrane with increasing concentration of the ion analyte in the solution. Then, the way to transform this strategy in an absolute analytical measurement is in combination with the thin-layer sample, balancing the capacity of the membrane with the charge in the solution, as herein proposed.

Effectively, for each K^+^ concentration, it was found that close to 100% of the ion concentration was transferred from the sample to the ISM. Table 1 presents these results, calculated considering the theoretical K^+^ concentration with which each sample was experimentally prepared (i.e., the recovery in % of the coulometry-based value compared to the theoretical one). In principle, this outcome suggested that it may be possible to calculate the K^+^ concentration in an unknown sample by just considering the charge under the corresponding voltammetric peak and without calibrating the electrode. Notably, higher values of % of absoluteness, and more deviated from the ideal 100%, were observed for the lowest K^+^ concentration (5 μM) within the measurement range. While an error of ca. 30% could appear when measuring a K^+^ concentration of 5 μM, this is significantly lower (5%) in the rest of the range. In any case, the absence of any temperature effect on the charge response confirmed the thin-layer behavior of the system.^12^ The charge under the K^+^ peak presented a variation of <1% when the temperature was varied from 10 to 40 °C, obtaining overlapping voltammograms for a selected concentration of 15 μM (data not shown).

**Table 1:** Differences in Percentage between the K^+^ Concentrations Calculated from the Experimental Cathodic Charges (via the Faraday Law) and Theoretical Ones (SD = Standard Deviation)

	% of absoluteness	
K^+^ concentration (μM)	at 5 mV s^–1^	at 1 mV s^–1^
5	135	111
10	112	104
15	101	101
20	99	102
25	100	103
30	98	97
40	97	96
average ± SD	101 ± 5^a^	102 ± 5^b^

a In the range from 10 to 40 μM.

b In the range from 5 to 40 μM.

It could be expected that decreasing the scan rate of the cathodic sweep even more would result in better shaped and separated peaks, being able to reduce even the limit of detection of the technique. Truly, at 1 mV s^–1^ (Figure S1a–c, Supporting Information), a slightly better separation of the Na^+^ and K^+^ peaks was found, but the improvement was not enough to justify the selection of a longer analysis time per sample (i.e., from a bit longer than 3 min to almost 17 min when using 5 and 1 mV s^–1^, respectively). Thus, the Na^+^–K^+^ peak separation was 165 mV on average at 5 mV s^–1^ and 185 mV at 1 mV s^–1^. Nonetheless, it seems more convenient to use a lower scan rate when K^+^ concentrations as low as 5 μM aim to be detected, since the method presents better efficiency at such a level (Table 1) and, hence, less error will be encountered. In any case, the average efficiency in the linear range of response was found to be quite similar at both scan rates (101% vs 102%, Table 1).

As in the case of 5 mV s^–1^, an excellent correlation between the experimental and theoretical K^+^ concentration in the sample was found at 1 mV s^–1^ (Figure S1d, Supporting Information): with a Pearson coefficient of 0.999. The variation of the peak current and charge for the three operated electrodes was on the order of the 5%, a bit higher than for 5 mV s^–1^, whereas the charge under the K^+^ peak displayed a variation of <2% when the temperature was varied from 10 to 40 °C (i.e., overlapping voltammograms for a selected K^+^ concentration of 15 μM, data not shown).

One interesting feature that appeared in the results at scan rates of 25, 5, and 1 mV s^–1^ is the presence of a kind of “interception point”, defined as a potential at which all the voltammograms at any K^+^ concentration present the same current level. The coordinates of such a point (absolute current in μA, potential in V) were found to be ∼(1.4, 0.5), (0.26, 0.5), and (0.025, 0.55) for 25, 5, and 1 mV s^–1^, respectively. In principle, the lowering in the current is expected as per decreasing the scan rate. Being that the potential is almost the same in the three cases, we believe that this is likely related to the selectivity of the ISM. Nevertheless, this hypothesis might be investigated later with other ionophores in the ISM.

Finally, the analytical potential of the developed calibration-free measurements was demonstrated by analyzing the K^+^ content in five water samples: seawater from the Fehmarn Belt Sea (sample 1, Puttgarden, Germany), seawater from the Mar Menor Sea (sample 2, Murcia, Spain), seawater from the Mediterranean Sea (sample 3, Alicante, Spain), lake water from the Vättern Lake (sample 4, Gränna, Sweden), and river water from the Lez River (sample 5, Montpellier, France). The samples were diluted with a 10 mM NaCl solution at ratios of 1:300, 1:1000, 1:1000, 1:5, and 1:100 for 1–5, respectively. The addition of NaCl was to ensure the conductivity of the samples, even when diluted. In addition, all the samples were analyzed with ion chromatography (IC). Samples 1–3 were diluted with ultrapure water at a ratio of 1:10, whereas samples 4 and 5 were directly measured.

Figure 4a–c depict the voltammograms observed for the five samples with three similar electrodes. Two peaks appeared in all the cases: the first peak at more positive potentials (ca. 650 mV) corresponds to K^+^, and the second peak (at ca. 450 mV) relates to other cations in the sample matrix. This latter mainly corresponds to Na^+^, which is the main cation present in the matrix. The three electrodes displayed very similar responses for each sample, while the K^+^ content was evidently different in all of them. Table 2 lists the observed K^+^ concentration in the samples with the developed coulometry approach (no calibration) and the IC (with a previous calibration). Overall, a good agreement was found with the IC technique, presenting differences of <10% for all the analyzed samples.

**Table 2:** Obtained K^+^ Concentrations in Five Different Water Samples Using the Developed Coulometry Approach and the Reference Method (IC)

sample	dilution	ISE (μM)^a^	IC (μM)	diff IC-ISE (%)
1	1:10		378.3	
1	1:300	12.9 ± 0.3	12.6^b^	2
2	1:10		1242	
2	1:1000	13.1 ± 0.3	12.4^b^	6
3	1:10		1094	
3	1:1000	11.3 ± 0.2	10.9^b^	3
4	0		45.3	
4	1:5	9.7 ± 0.2	9.1^b^	7
5	0		81.6	
5	1:10	8.8 ± 0.3	8.2^b^	8

a Average ± SD (*n* = 3 electrodes).

b The concentrations were calculated considering the dilution factors.

**Figure 4:**
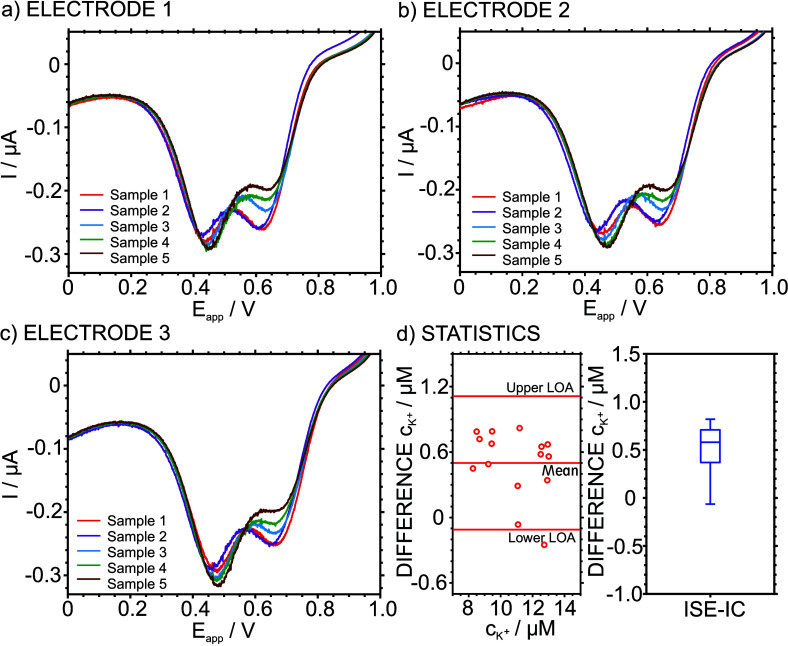
(a–c) Cathodic peaks observed for samples 1–5 at a scan rate of 5 mV s^–1^ using three identical ITO-POT-membrane electrodes. (d) Bland–Altman and box plots.

Figure 4d shows the Bland–Altman and box plots, with the latter based on the differences between the two methods. Assessing first the agreement between the methods (Bland–Altman plot), a percentage bias of 5% was found, indicating that the developed calibration-free method slightly overestimated the K^+^ concentration in the samples. Most of the data points (except one sample) fitted in the 95% limit of agreement, meaning that the differences between the two methods are relatively null. The median value (box plot) was 0.58 μM, with a small interquartile range (0.38 μM), suggesting that the differences between the two methods are at similar levels among the samples. Importantly, no statistically significant differences were found between the charge-based readout and IC.

Altogether, a coulometry calibration-free methodology for the determination of K^+^ using all-solid-state ISEs has been herein demonstrated. The accumulation of K^+^ from the thin-layer sample to the ISM was clearly visualized as a cathodic peak that can be treated chargewise for analytical purposes. There is yet room for development of the concept presented in this Letter. On one hand, the accuracy may be further improved by even more fine control of the sample volume, restricting the lateral diffusion in the microfluidic channel and broadening the range of response by investigating the ISM composition and electrochemical protocol for the measurements (e.g., chronocoulometric readout). Notably, any change in the cation exchanger concentration (indeed, the charge) will involve a recalculation of the cell dimensions, changing the sample volume while providing the thin-layer sample perspective. Additionally for the ISM composition, the significance of the developed approach also relies on the possibility of providing coulometric measurements of more than one ion in only one cathodic sweep, i.e., owing to the incorporation of several ionophores. Indeed, the application of electrochemical protocol based on “chrono” methods rather than linear sweep potential is expected to be beneficial selectivity-wise.

## Supporting Information Available

Experimental section and voltammograms at 1 mVs^–1^ (PDF)
