# Supplementary material for: Approaching to Calibration-Free Ion Detection Based on Thin Layer Coulometry with Ultrathin Ion-Selective Membranes
Source: ACS Meas Sci Au. 2024 Dec 10;5(1):63–9. doi: 10.1021/acsmeasuresciau.4c00069 (PMC11843505; doi:10.1021/acsmeasuresciau.4c00069)
Supplement: Supplementary file 1 — tg4c00069_si_001.pdf [file tg4c00069_si_001.pdf]

Supporting Information for:

Approaching to Calibration-Free Ion Detection based on Thin Layer Coulometry with Ultrathin Ion-selective membranes

Yujie Liu[1], Gastón A. Crespo[1,2], María Cuartero[1,2,*]

1Department of Chemistry, School of Engineering Science in Chemistry, Biochemistry and Health, KTH Royal Institute of Technology, SE-100 44 Stockholm, Sweden
2UCAM-SENS, Universidad Católica San Antonio de Murcia, UCAM HiTech, Avda. Andres Hernandez Ros 1, 30107 Murcia, Spain

Experimental Section

Reagents, materials, and equipment. Aqueous solutions were prepared by dissolving the appropriate salts in deionized water (>18.2 MΩ). 3-octylthiophene (97%, OT), lithium perchlorate (>98%, LiClO4), polyurethane (PU, Selectphore), high molecular weight poly(vinyl chloride) (PVC), bis(2-ethylhexyl)sebacate (DOS), sodium tetrakis[3,5-bis-(trifluoromethyl)phenyl]borate (NaTFPB), potassium ionophore I (Valinomycin), sodium chloride (99.999%, NaCl), potassium chloride (99.5%, KCl), tetrahydrofuran (>99.9%, THF) and acetonitrile (anhydrous, >99.8%, ACN) were purchased from Sigma Aldrich. Absolute ethanol (99.5%, CH_3CH_2OH) was acquired in VWR. Indium tin oxide (ITO) coated glass slides (10 mm × 35 mm × 1.1 mm, surface resistivity <10 Ω/sq, transmittance >83%) were sourced from Zhuhai Kaivo Optoelectronic Technology. Screen-printed platinum electrodes (Pt-SPE, DRP-550) were purchased from Metrohm Dropsens. The PTFE and the copper tapes were purchased from RS Components. The silicon rubber was supplied by Junying CNClathing company.

Calculations were accomplished in MATLAB_R2023a software. Cyclic voltammetry (CV) measurements were performed using a VIONIC potentiostat controlled by INTELLO software supplied by Metrohm. An 850 Professional Ion Chromatography (IC) instrument equipped with a Metrosep C6−150/4.0 column, conductivity detector, and 863 Compact Autosampler (injection volume of 10 µL) sourced from Metrohm was used to validate the real sample analysis (sample eluent of 2.5 mM HNO_3, flow rate of 0.9 mL/min).

Preparation of the ITO-POT-membrane electrode. ITO electrodes were cut to the size of 10 mm × 35 mm. The pieces were cleaned with ethanol via ultra-sonification, and then rinsed with water. A PTFE tape shaped with a circle was applied to the ITO surface to define a surface with a diameter of 8 mm in where to electropolymerized a thin layer of POT (thickness ~50 nm, measured with ellipsometry). Later, the tape was carefully removed to deposit the nanosized membrane (thickness of ~230 nm, measured with ellipsometry) on top of the ITO-POT.

For the modification of the ITO, as well as for subsequent electrochemical measurements, an electrical contact was established in one of the ITO corners by using a copper tape. The electropolymerization of the POT film on the ITO was carried out by CV in a solution containing 0.1 M of 3-octylthiophene and $LiClO_4$ in ACN. Briefly, after degassing with nitrogen for 15 minutes, 2 CV scans within the potential window from of 0 to 1.5 V at a scan rate of 100 mV/s were performed. The generated POT film was then discharged at 0 V for 120 s to ensure that most of the POT chains are in its neutral state (POT^0), which is the most stable one for the polymer. A platinum rod (6.1248.000, Metrohm) and a home-made Ag/AgCl wire were used as the counter and reference electrodes in the eletropolymerization procedure. Notably, the electrochemical cell used for the POT synthesis and discharging was reported elsewhere. Then, the ITO-POT electrode is disassembled from the cell and immersed first in ACN and after that in THF for 30 minutes and 10 seconds, respectively, being finally dried with a smooth flow of compressed air.

The cocktail of PU-based membrane was formulated by mixing 20 mg of PU, 20 mg of DOS, 0.8 mg of NaTFPB, and 2 mg of potassium ionophore I in 2 mL of THF. Finally, the membrane was deposited onto the POT-ITO electrode by spin coating 25 µL of the membrane cocktail (1500 rpm, 60 s) using a 6808P spin coater provided by PI-KEM.

Samples. The following samples were analyzed: sea water from Fehmarn Belt Sea (Sample 1) Puttgarden, Germany; sea water from Mar Menor Sea (Sample 2) San Javier, Spain; sea water from Mediterranean Sea (Sample 3) Torrevieja, Spain; lake water from Vättern lake (Sample 4) Gränna, Sweden; and river water from Lez River (Sample 5) Montpellier, France. The samples were filtered firstly by regular filter paper (manufactured by Whatman™ with a diameter of 150 mm), and then by 0.2 μm pore-size filters to remove large particles. Sample 1-5 were diluted with a 10 mM NaCl solution at ratios of 1:300, 1:1000, 1:1000, 1:5, and 1:100, respectively. The addition of NaCl is to ensure the conductivity of the diluted samples. In IC measurement, Sample 1-3 were filtrated and then diluted with ultra-pure water at a ratio of 1:10. Sample 4 and 5 were directly measured after filtration without dilution.

Figures

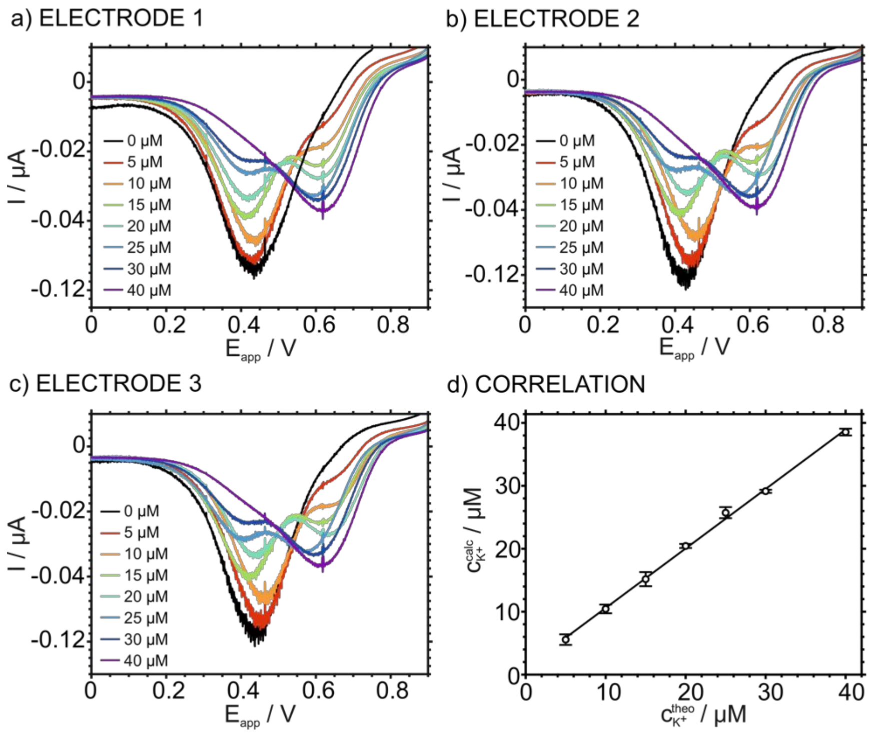

Figure S1. (a-c) Cathodic peaks observed at increasing concentrations of KCl in 10 mM NaCl background solution at the scan rate of 1 mV s^{-1} using three identical ITO-POT-membrane electrodes. (d) Correlation between the calculated and theoretical K^+ concentration in the sample solution (n=3).
